# Confusion Worse Confounded

**Published:** 1890-01-04

**Authors:** 


### The Doctor's Pulpit.

### "CONFUSION WORSE CONFOUNDED."

It is plain even to an observant child that all the varieties of human beings belong to the same order of creatures. It is equally undeniable that under the same or similar conditions all sorts of human beings will develop the same or similar peculiarities. Looking at man, for example, as the subject of what we call "disease," it is clear that this peculiarity of his experience must assume certain forms, but forms that are strictly limited in range and variety. As a matter of fact, we find that the essential varieties of diseased processes are very few, and that all the multitudinous disorders which affect mankind may be grouped under an exceedingly limited number of heads. Taking inflammation as the parent of more diseases and disorders than any other physiological or pathological process known, is it not obvious that inflammation is always inflammation, and never anything else but inflammation? It may be of the brain or of the finger; it may be of the bowel surrounding a typhoid ulcer, or of the throat surrounding and underlying a patch of membranous exudation; it may be of the skin modified by eczema, erysipelas, or scarlet fever; or it may be of the mucous membrane specialised and dominated by one of a dozen blood poisons. But inflammation is always an inflammatory process, and, however its treatment be modified and specialised, it must still be a treatment appropriate to inflammation.

But if practically all diseases may be classed under a very limited number of leading groups, it is obvious that their treatment may also be brought within the action of a limited range of principles. What is meant is that there must be certain methods of doing things in the medical art, and that experience either has, or ought to have, found out the very best. But as the things to be done are in their essential nature very few in number, so the best methods of doing them are very few in number. But as these best principles of treatment are few in number, they are correspondingly easy to master, and therefore we have a right to expect and insist that they shall be universally recognised, understood, and practised, throughout the whole of the medical profession. In other words, there are principles of medicine exactly as there are principles of law, of theology, of engineering, of book-keeping, and of every other art; and the neglect of these principles or the failure to understand them clearly is productive of the same kind of disaster in medicine as overtakes the engineer who does not apply the principles of engineering in his practice, or the builder who throws away his plummet and his spirit-level when he goes to work.

But what are the facts in the actual practice of the medical art to-day? Are the leading principles of practice always and everywhere borne in mind and carried out? It is much to be feared that they are not. It may certainly be said of the methods of medical treatment that they are, like Cleopatra, full of "infinite variety." And it cannot be denied that as a consequence they are too often characterised by infinite inefficiency. Can any medical man within the whole range of his recollection recall two identical prescriptions for identical diseases given by different physicians? No doubt there is often a good deal of similarity, but the extraordinary diversity of drugs prescribed for the same conditions by different doctors is absolutely startling. It is not that medical men are ignorant of diseases and their pathological history; nor yet is it that they do not understand the physiological action of remedies. But it is that each man approaches his case from his own particular standpoint alone, and not as influenced by great principles and dominating minds. There has been for a long time a remarkable dearth of influential teaching intellects in the medical profession; and concurrently with this there has been a period of extraordinary discovery of isolated facts. These facts have been thrown at random into the general mass of medical knowledge; and their effect has been to produce scepticism, confusion, and an altogether disheartening lack of efficiency and success.

No student of history can for a moment doubt the paramount and invaluable influence of clear and commanding intellects. Whether the field of politics be chosen for observation or that of morals, or of law, the conclusions are the same. The average man, even of the educated classes, does not possess sufficient originality of mind, industry, and clear sightedness to push his way to the best knowledge and to the mastery of the best principles. He needs a guide; a guide who can arouse his enthusiasm, convince his intelligence, and inspire his faith. With such a guide he will lay hold of great principles, he will believe in them, he will apply them in practice. But where is that guide to be found among the physicians and surgeons of the last twenty years? And where, in the English-speaking world, is his platform, supposing he were found? We do not say there are no medical intellects capable of acting as guides; but we do say that those who could have acted in that capacity, and who would have so acted if their professional enthusiasm had been stronger, have, practically without exception, yielded to the temptation to seek wealth and worldly honour in the obscure recesses of the consulting-room.

It is more than unfortunate that the medical profession offers few or no opportunities to men of the highest teaching capacity. This is the case all throughout England, and conspicuously so in London. It is remarkable that the University of Edinburgh, among all the medical schools of Great Britain, alone offers anything like an adequate reward to the pure teacher. The medical lectureships of the London hospitals are mere ladders whereby comparatively young men climb to the prosperous dignity of the private consulting-room. There is no Parnassus of medical lectureships, and, hence, no medical lecturer is ever, as a lecturer, crowned with dignity and honour.

The consequences of this state of things are disastrous all round. Medical literature is, as a result, always ephemeral and often contemptible. The leaders of medicine produce nothing whereby the contemporary public and posterity can compare their intellectual quality with that of men of distinction in other walks of life. No medical man takes any pride in the well-earned honours and commanding influence of other medical men. Every man, feeling secure from general criticism, fights unceasingly for his own hand. The practice of the medical art throughout the country, being based on no great principles, is uncertain, chaotic, feeble, and discreditable. Just as no country can ever become great without great intellects and great stages on which they can play their parts, so will the medical profession never rise above its present level of half-comprehended mediocrity until it seeks out from its ranks teachers who can teach, gives them positions in which their teaching can be effective, and crowns them with the full honours due to the greatness and permanent value of their work.

